# Knockout rat models mimicking human atherosclerosis created by Cpf1-mediated gene targeting

**DOI:** 10.1038/s41598-019-38732-2

**Published:** 2019-02-22

**Authors:** Jong Geol Lee, Chang Hoon Ha, Bohyun Yoon, Seung-A. Cheong, Globinna Kim, Doo Jae Lee, Dong-Cheol Woo, Young-Hak Kim, Sang-Yoon Nam, Sang-wook Lee, Young Hoon Sung, In-Jeoung Baek

**Affiliations:** 1 ConveRgence mEDIcine research cenTer (CREDIT), Asan Institute for Life Sciences, Asan Medical Center, Seoul, Republic of Korea; 2 Biomedical Research Center, Asan Institute for Life Sciences, Asan Medical Center, Seoul, Republic of Korea; 3 College of Veterinary Medicine, Chungbuk National University, Cheongju, Republic of Korea; 4 Department of Convergence Medicine, University of Ulsan College of Medicine, Asan Medical Center, Seoul, Republic of Korea; 5 Department of Cardiology, University of Ulsan College of Medicine, Asan Medical Center, Seoul, Republic of Korea; 6 Department of Radiation Oncology, University of Ulsan College of Medicine, Asan Medical Center, Seoul, Republic of Korea

## Abstract

The rat is a time-honored traditional experimental model animal, but its use is limited due to the difficulty of genetic modification. Although engineered endonucleases enable us to manipulate the rat genome, it is not known whether the newly identified endonuclease Cpf1 system is applicable to rats. Here we report the first application of CRISPR-Cpf1 in rats and investigate whether *Apoe* knockout rat can be used as an atherosclerosis model. We generated *Apoe-* and/or *Ldlr*-deficient rats via CRISPR-Cpf1 system, characterized by high efficiency, successful germline transmission, multiple gene targeting capacity, and minimal off-target effect. The resulting *Apoe* knockout rats displayed hyperlipidemia and aortic lesions. In partially ligated carotid arteries of rats and mice fed with high-fat diet, in contrast to *Apoe* knockout mice showing atherosclerotic lesions, *Apoe* knockout rats showed only adventitial immune infiltrates comprising T lymphocytes and mainly macrophages with no plaque. In addition, adventitial macrophage progenitor cells (AMPCs) were more abundant in *Apoe* knockout rats than in mice. Our data suggest that the Cpf1 system can target single or multiple genes efficiently and specifically in rats with genetic heritability and that *Apoe* knockout rats may help understand initial-stage atherosclerosis.

## Introduction

The laboratory rat is a valuable experimental model organism and has greater translational relevance than the mouse due to its greater similarity to humans in many biological aspects^1,2^. Its larger size compared to mice enables various interventional procedures such as surgery and high-resolution imaging, and its rich behavioral profile is beneficial in neuroscience research^3^. Despite its advantages, genetically engineered rat models have been extremely limited in number^4^. In the past, mice have been preferred as mutant animal model than rats as the rat ES cell is less robust than mouse ES cell and public resources for mutant mice are plentifully available^5^. Nowadays, genome editing technology is rapidly being advanced due to the development of engineered endonucleases such as zinc-finger nucleases (ZFN), transcription activator-like effector nucleases (TALENs), and the clustered, regularly interspaced, short palindromic repeat (CRISPR) systems, and the first applications of ZFN^6^, TALEN^7^, and CRISPR/Cas9^8,9^ to target rat genome have been reported continuously along with the emergence of next-generation engineered endonucleases. CRISPR from *Prevotella and Francisella 1* (Cpf1) is a new type V CRISPR-Cas endonuclease^10^. With distinct features different from the former CRISPR/Cas9, Cpf1 is recently known to be efficient in genome editing in plant cells^11^, *Drosophila*^12^, human cells^10^, and mice^13,14^. However, until now, its application has not yet been proved in rats.

Cardiovascular disease, a leading cause of mortality in industrialized countries, is caused mainly by atherosclerosis. Atherosclerosis is primarily initiated by the imbalance of blood lipids^15^, and apolipoprotein E (*Apoe*) plays a pivotal role in transport and metabolism of the cholesterol-rich lipoproteins^16^. Its deficiency contributes to the risk of type III hyperlipoproteinemia (HLP III) and familial dysbetaliproteinemia, and consequently higher risk of atherosclerosis^17,18^. Mice lacking *Apoe* gene have been the most widely used animal model for atherosclerosis^19^ since the first *Apoe* KO mice were produced by classical gene knockout strategy using homologous recombination in ES cell with targeting vector^20,21^. Low density lipoprotein receptor (*Ldlr*) also removes cholesterol-rich lipoproteins from plasma thereby to regulate the blood cholesterol level^22^, and *Ldlr* knockout mice^23^ are another preferred animal model for atherosclerosis, although the use of *Ldlr*^−/−^ mice is less frequent than *Apoe*^−/−^ mice^24^.

Despite that mice deficient in either *Apoe* or *Ldlr* have expanded our understanding in atherosclerosis, the translation from mouse to human has not been so persuasive in the field of cardiovascular research including atherosclerosis. In terms of drug development, the success rates of clinical trials for cardiovascular disease are among the lowest of all major medical fields^25^. This phenomenon may arise in part from the fact that atherosclerosis in mice is quite different from that in humans. Particularly, in contrast to human atherosclerosis which develops very slowly^4^, short progression time in *Apoe* deficient mice renders it unclear how atherosclerosis initiates at early stage. Meanwhile, the rat is a widely accepted model animal for cardiovascular research, particularly for hypertension and stroke^3^. So far, there has been only a small number of papers suggesting *Apoe* or *Ldlr* knockout rat as an atherosclerosis model which were generated with engineered endonucleases: TALEN-mediated *Apoe* knockout^26^, ZFN-mediated *Ldlr* knockout^27^, and CRISPR/Cas9-mediated knockout of *Apoe* or *Ldlr*^28^ in rats. The *Apoe* or *Ldlr* deficient rats showed some phenotypic variations in atherosclerosis according to type of deficient genes and the way to induce atherosclerosis (eq. high-fat diet (HFD) duration, artificial induction of endothelial injury, etc.), but the previous reports have been focused on phenotyping typical atherosclerosis at later stage which is classically represented by plaque formation in mice. Considering that there is no appropriate genetically modified animal model for early atherogenesis^29^ and previously known progression of atherosclerosis in *Apoe* knockout rat is relatively slower than that of mouse, it could be speculated that *Apoe* knockout rats could be more suitable preclinical animal model to reproduce the normal or pathological background of early stage atherosclerosis in humans.

Herein, we investigated whether the recently identified engineered nuclease Cpf1 system^10^ could be used to manipulate the rat genome *in vivo*. In addition, we investigated whether Cpf1-mediated *Apoe* knockout rat could serve as an animal model to mimic the progression of human early atherosclerosis.

## Results

### Cpf1-mediated generation of *Apoe* and/or *Ldlr* knockout rats

**Figure 1:**
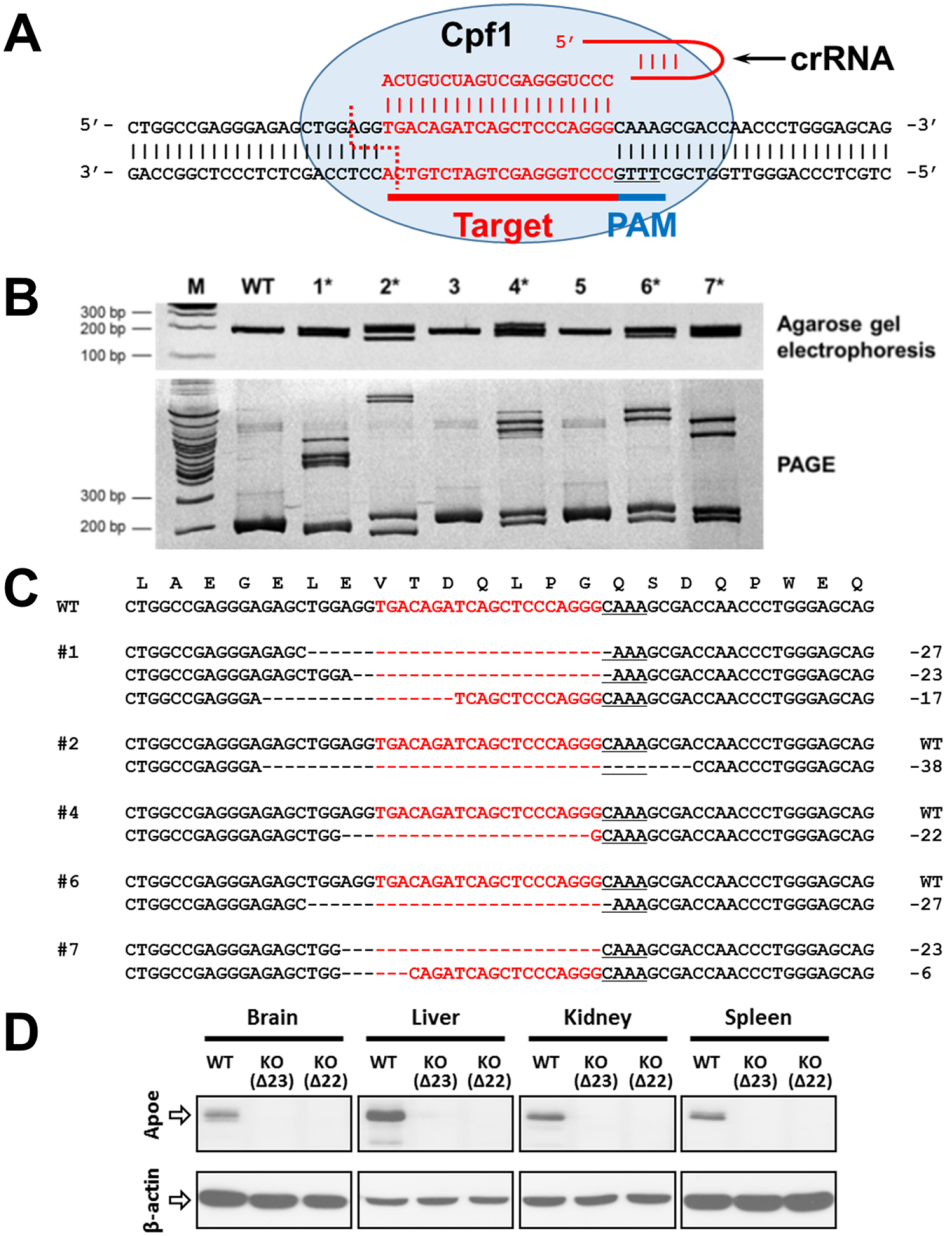
Generation of Cpf1-mediated *Apoe* mutant rats. (**A**) Target DNA sequence in the rat *Apoe* locus is shown in red. This region corresponds to the nucleotides 185–250 of the *Apoe* transcript variant 1 mRNA (NM_001270681.1) encoding amino acids 17–38 of the rat apolipoprotein E precursor protein (NP_620183.2). Dotted line marks the potential staggered cut possibly generated by the Cpf1-crRNA pair. The underlined sequence represents the PAM. (**B**) Agarose gel electrophoresis- and PAGE-based genotyping assays identifying founder rats derived from pronuclear-stage embryos intra-cytoplasmically injected with *LbCpf1* mRNA (50 ng/μL) and its cognate crRNA (100 ng/μL) targeting the rat *Apoe* locus. The numbers denote each newborn rat; M, molecular size marker; WT, wild-type rat; *, founder rat. The full-length gels are presented in Supplementary Fig. S9. (**C**) Mutated *Apoe* sequences observed in the founder rats in B. “–” denotes deleted nucleotides. (**D**) No detectable Apoe protein in *Apoe*-deficient rats. *Apoe*-mutant founders (F0) #7(∆23/∆6) and #4(∆22) generated with LbCpf1 were each crossed with WT rats, and the heterozygous F1 from founders were intercrossed to generate wild-type (WT) or homozygous knockout (KO) rats. The levels of Apoe protein expression in the brain, liver, kidney, and spleen from *Apoe* KO rats were then compared to the expression in corresponding tissues of WT rats. The full-length blots are presented in Supplementary Fig. S10.

To examine whether Cpf1 is suitable for gene targeting in rats, we used the Cpf1 ortholog from *Lachnospiraceae bacterium N D2006* (LbCpf1) and generated a CRISPR RNA (crRNA) targeting exon 3 of the rat *apolipoprotein E* (*Apoe*) gene (*Apoe-crRNA)*, which is common among the rat *Apoe* transcript variants (Fig. 1A). Similar to the procedure used for mice in our previous study^30^, *LbCpf1* mRNA (50 ng/μL) and *Apoe-crRNA* (50 or 100 ng/μL) were simultaneously microinjected into the cytoplasm of pronuclear-stage embryos of Sprague–Dawley (SD) rats; then, the embryos were transferred into the oviduct of pseudo-pregnant foster mothers. There was no sign of acute toxicity immediately after the microinjection, but only a small fraction of live newborns were obtained. PAGE-based genotyping analyses identified seven mutants (38.9%) out of 18 newborns (Table 1, Fig. 1B, and Supplementary Fig. S1A). Injection of the 100-ng/μL *Apoe-crRNA* dose yielded 5/7 mutant founder (F0) rats (71.4%; Table 1 and Fig. 1B), and a lower dose of *Apoe-crRNA* (50 ng/μL) yielded 2/11 F0 mutants (18.2%; Table 1 and Supplementary Fig. S1A), suggesting a dose-dependent effect of *Apoe-crRNA*. Sanger sequencing analyses showed that all mutations were deletions ranging from 6 to 38 base pairs (bp) (Fig. 1C and Supplementary Fig. S1B). Contrary to the results of the previous study in mice^30^, mosaicism was not frequently observed (1 out of 7 F0 rats; Fig. 1C and Supplementary Fig. S1B). The functional disruption of the APOE protein by loss of the *Apoe* gene was further confirmed by the absence of APOE protein expression in *Apoe*-deficient rat tissues (Fig. 1D).

**Table 1:** Nuclease activity of LbCpf1 in Sprague–Dawley rat embryos and generation of *Apoe* gene knockout rats.

crRNA + Cpf1 mRNA (ng/μL)	Injected zygotes	Survived & transferred (%)^a^	Newborns (%)^b^	Mutants (%^b^, %^c^)	Mosaicism frequency (%)^d^
100 + 50	42	41 (97.6)	7 (17.1)	5 (12.2, 71.4)	1 (20)
50 + 50	77	77 (100)	11 (14.3)	2 (2.6, 18.2)	0 (0)

Percentages were calculated using the number in each column as the numerator and the numbers of ^a^injected zygotes, ^b^surviving and transferred embryos, ^c^newborns, or ^d^mutants as the denominator.

Efficient germline transmission of mutations is required to establish genetically modified rat models. To investigate the germline transmission of Cpf1-mediated gene mutations, we crossed *Apoe* founders F0 #4 and F0 #7 with WT rats, and the genotypes of their pups were determined by Sanger sequencing. The mutant alleles identified in the founders were found to be successfully germline transmitted (Supplementary Fig. S2).

We also produced *Ldlr* knockout rats by targeting the fourth exon of the rat *Ldlr* gene, in this case using a Cpf1 ortholog from *Acidaminococcus sp. BV3L6* (AsCpf1) (Supplementary Fig. S3A). We obtained 22 newborns after microinjecting 63 zygotes (34.9%) and identified 12 F0 mutants (54.5%; Supplementary Fig. S3B and Supplementary Table S1). Notably, mosaicism was frequently observed (six mosaic mutants out of 12 F0 rats; Supplementary Fig. S3C), suggesting that the occurrence rates and characteristics of Cpf1-induced mutations vary depending on the target site. When the mosaic F0 #13 (WT/∆30/∆50/∆57/∆64) was crossed with WT, all of the mutant alleles were inherited by the F1 progeny (Supplementary Fig. S4), demonstrating that this Cpf1-mediated gene targeting system is a useful tool for generating rats with heritable genetic changes.

One of the most important advantages of Cpf1 is that it can be guided by crRNAs targeting multiple genomic loci simultaneously with low toxicity in rat embryos, as previously reported in mouse embryos^30^ and mammalian cells^31^. Given the high mutation rates induced by Cpf1 orthologs in rats, we tested whether multiple loci could be targeted simultaneously in rats. We targeted the *Apoe* and *Ldlr* genes simultaneously and observed that rat embryos contained mutations in both genes (4 out of 7 embryos; Supplementary Fig. S5 and Supplementary Table S2), suggesting that the Cpf1-based gene targeting system is also useful in other situations for generating multiple gene knockout rats.

*In vivo* specificity is another important issue in Cpf1-mediated genome editing. To determine the specificity of the Cpf1 orthologs, possible off-target sites with 3- to 4-bp mismatches with the target sequences were analyzed (Online Supplementary Tables 3 and 4). We could find no remarkable off-target effects in *Apoe*-mutated (Supplementary Fig. S6A) or *Ldlr*-mutated founders (Supplementary Fig. S6B), which is in agreement with previous reports stating that Cpf1 is highly specific, with few or no off-target effects in mice^30^ or human cell lines^13,31^.

Thus, these results clearly prove that Cpf1 can be a highly efficient tool for generating knockout rats.

### Cpf1-mediated *Apoe* knockout rat as a potent animal model for initial stage atherosclerosis

**Figure 2:**
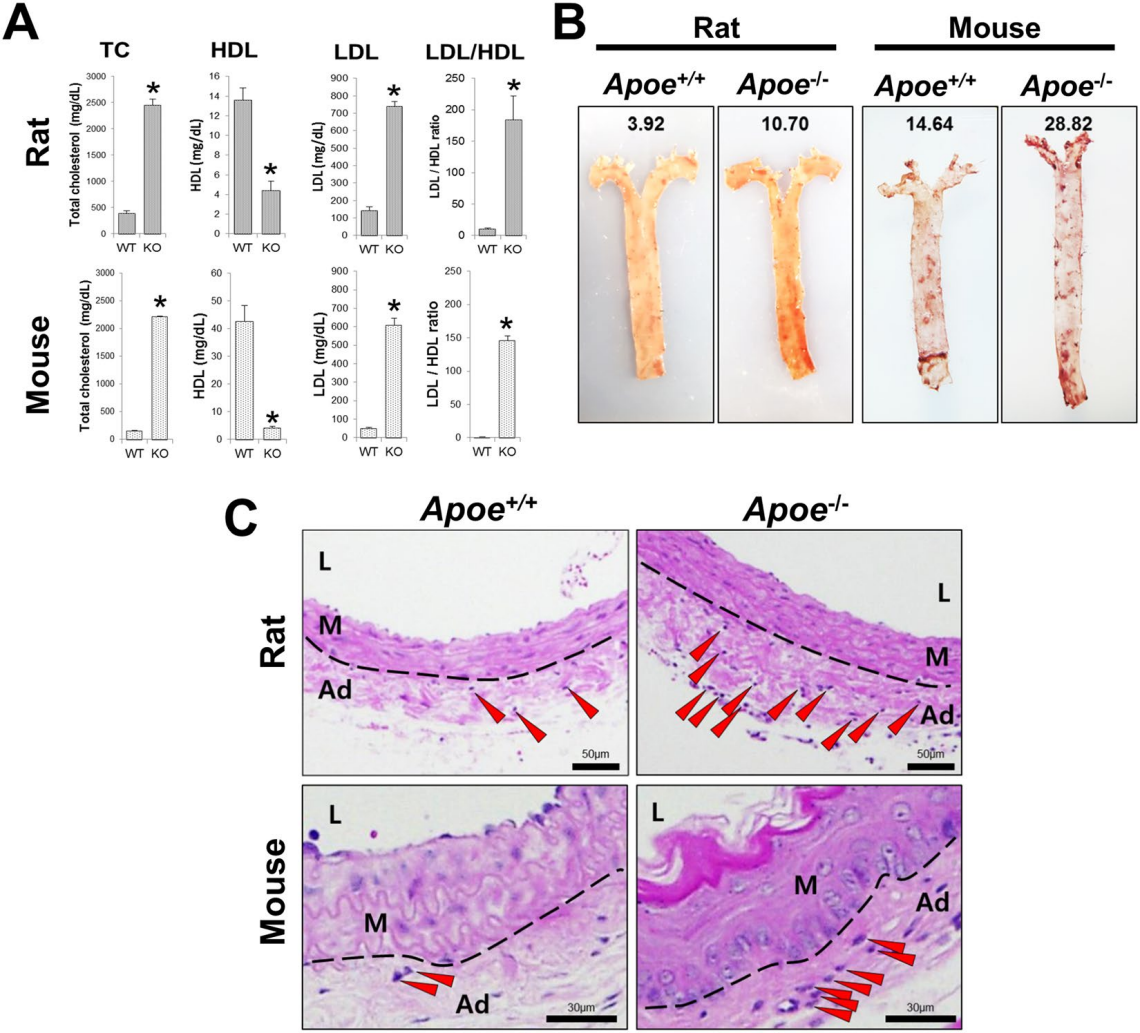
*Apoe* knockout rats developed proatherogenic lesion of earlier stage than *Apoe*-deficient mice in partially ligated carotid artery. (**A**) Hypercholesterolemia in *Apoe* knockout rats and mice. Serum levels of total cholesterol (TC), high-density lipoprotein (HDL), and low-density lipoprotein (LDL), as well as the LDL/HDL ratio, were measured in 8 to 10 week-old WT and *Apoe*-deficient animals fed with a high-fat diet (HFD) for 2 weeks. Data are expressed as mean ± standard errors of the means. Asterisks mark statistically significant differences from the WT group (P < 0.001 using one-way ANOVA; n = 3/genotype). (**B**) Atherosclerotic aortic lesions in *Apoe* knockout rats and mice. Aortae from WT and *Apoe*-deficient animals with HFD fed for 2 weeks were stained with oil red O (ORO). The lesion areas were expressed as percentages of lesions relative to the surface area of the entire aorta. (**C**) H&E staining of partially ligated carotid artery from *Apoe* knockout rats and mice. Red arrowheads indicate immune infiltrates in adventitia. L, lumen of carotid artery; M, media; Ad, adventitia.

To determine the functional consequences of *Apoe* gene disruption and the viability of Cpf1-mediated *Apoe* knockout rats as an atherogenic model, wild-type (WT) and *Apoe*-deficient rats were fed a HFD, and their serum lipids were analyzed. HFD-fed *Apoe*-deficient rats displayed hyperlipidemia with dramatically elevated levels of serum cholesterol and low-density lipoprotein (LDL) compared to those of WT rats (Fig. 2A, upper panel), which sustained similar levels even until 8 weeks of HFD feeding (data not shown). *En face* whole aortae from HFD-fed WT and *Apoe*-deficient rats were stained with oil red O (ORO), revealing that the atherosclerotic lesions were more developed in aortae from *Apoe* knockout rats than in those from WT rats (Fig. 2B, left panel). Moreover, ORO-stained lipidic areas were abundant in coronal sections of carotid artery from *Apoe*-deficient rats, whereas in sections from WT rats, they were not abundant and were found mostly in the adventitia (Supplementary Fig. S7), which are involved in the early stages of the atherosclerotic process^32^.

Intimal thickening or neo-intimal formation was not found in *Apoe* knockout left carotid artery (LCA) at 2 weeks after partial ligation with HFD feeding, and the thickness of arterial wall remained normal (Fig. 2C, upper panel). This section contained no lipid-loaded macrophage (foam cell), which is known to appear in the early stages of atherosclerosis^33^, and even endothelial continuity was well preserved. However, notably, the only histologically different feature was the presence of increased immune infiltrates in the adventitia of *Apoe* knockout LCA as compared with that of unligated right carotid artery (RCA); this feature was not seen in WT rats (Fig. 2C, upper panel). In contrast to *Apoe* knockout mice, which showed robust neointimal formation after partial ligation with HFD, finally leading to almost stenotic lumen filled with plaques^34^, *Apoe* knockout rats in this study showed relatively slower progression of atherosclerosis, suggesting the possible inter-species variation in the developmental rate of atherosclerosis.

To determine the differences between mice and rats as an animal model inducing atherosclerosis, we also made *Apoe* knockout mice and compared the rate of progression of atherosclerosis between *Apoe* knockout rat and mice. Mutant mice having 13 bp deletion in exon 2 of the *Apoe* gene (nucleotide 216–229 of the *Apoe* transcript variant 1 mRNA (NM_009696.4)) were generated by CRISPR/Cas9 system, and their heterozygous founders were intercrossed to obtain WT and *Apoe* homozygous knockout mice (data not shown). Similarly to our observations in *Apoe* knockout rats (upper panel in Fig. 2A and left panel in Fig. 2B), severe hyperlipidemia and aortic lesions were both found in *Apoe*-deficient mice as compared to WT mice (lower panel in Fig. 2A and right panel in Fig. 2B). In partially ligated carotid artery of fed artery of fed with HFD for 2 weeks, atherogenic features indicating its intermediate stage, such as atherosclerotic debris within the lumen and degenerative changes in the media along with its reduced structural integrity, were detected in *Apoe* knockout mice (Fig. 2C, right side in lower panel), whereas these aspects of WT mice remained relatively normal. Particularly, the number of adventitial infiltrates increased in *Apoe*-deficient carotid artery (Fig. 2C, lower panel), which was also found in *Apoe*-deficient rats (Fig. 2C, upper panel).

**Figure 3:**
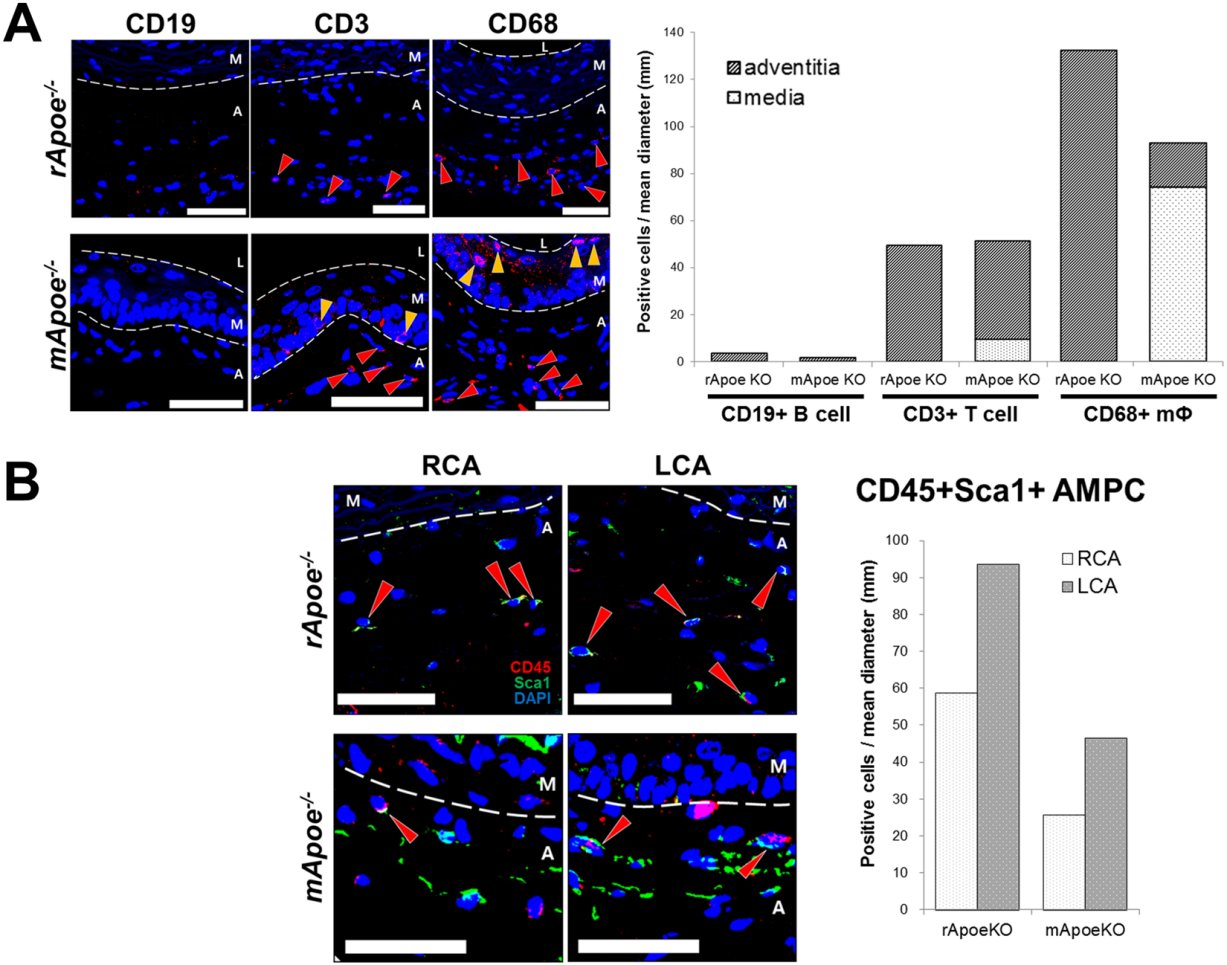
Different immune cell distribution between *Apoe* knockout rats and mice in partially ligated carotid artery. *Apoe*-deficient animals with 8 to 10 week-old age were undergone partial ligation of left carotid artery and subsequently fed with HFD for 2 weeks. (**A**) CD19-, CD3-, and CD68-positive cells in partially ligated carotid artery from *Apoe* knockout rats and mice. Arrowheads indicate positive cells in media (yellow) or adventitia (red). Scale bar, 50 μm. (**B**) Adventitial macrophage progenitor cells (AMPCs) in arteries of *Apoe* knockout rats and mice. Red arrowheads indicate CD45^+^Sca1^+^ cells. RCA, un-ligated right carotid artery; LCA, left partially ligated carotid artery; M, media; A, adventitia. Scale bar, 50 μm.

Next, based on the similarities (increased adventitial immune infiltrates) and differences (progression in atherosclerosis stage) between *Apoe*-deficient mice and rats, we speculated that the slower progression of atherosclerosis in *Apoe* knockout rats than in mice could be attributed to different immune population. Thus, immune cells within the ligated arterial wall were compared between the two species numerically and spatially, with a specific focus on macrophages, T cells, and B cells, which are reportedly the most abundant immune cells in atherosclerotic arteries of *Apoe* knockout mouse^35,36^. In partially ligated LCA of both *Apoe*-deficient rats and mice, although B cells were rarely detected (3.5 and 1.5 C/md in rat and mouse, respectively; Fig. 3A), the number of T cells was comparable between the two species (49.4 and 51.1 C/md in rat and mouse, respectively; Fig. 3A), and macrophages were the most abundant among the three immune cells (Fig. 3A). Notably, macrophages in the LCA of *Apoe*-deficient mouse were fewer than those of rat (132.3 and 92.9 C/md in rat and mouse, respectively; Fig. 3A) and were detected mainly in media rather than adventitia (74.3 and 18.6 C/md in media and adventitia, respectively; Fig. 3A). Conversely, macrophages in *Apoe* knockout rat were found only in adventitia (Fig. 3A). The larger medial macrophage distribution in mice than in rats may be due to their more progressed atherosclerotic stage, which is in accordance with previous reports stating that macrophages are abundant in media in intermediate-stage atherosclerosis^37^ and medial smooth muscle cells can transdifferentiate into macrophage-like cells, thus contributing to neointimal formation under atherosclerotic conditions^38–40^.

To understand the higher number of adventitial macrophages in *Apoe*-deficient rats than in mice, we then sought adventitial macrophage progenitor cells (AMPCs), which are resident in adventitia, to act as a source of macrophages^41^. AMPCs were originally present in hyperlipidemic arterial adventitia both in rat and mouse (Fig. 3B), which is in agreement with the previous report using *Apoe*-deficient mouse^41^. AMPCs were more abundant in the LCA than in the RCA, which was seen both in *Apoe*-deficient rats and mice (in rat, 58.6 and 93.5 C/md in RCA and LCA, respectively; in mouse, 25.7 and 46.5 C/md in the RCA and LCA, respectively; Fig. 3B), indicating that the number of arterial AMPCs was increased by atherogenic event in rats and mice. Interestingly, although atherosclerosis development was more progressed in *Apoe* knockout mouse than in rat (Fig. 2C), AMPCs of *Apoe* knockout rat in hyperlipidemic artery outnumbered those of mouse (Fig. 3B), which may have contributed to the higher adventitial macrophage population in *Apoe*-deficient rat than in mouse.

Collectively, these results suggest that Cpf1-mediated *Apoe* knockout rat could be a better model for initial-to-early atherosclerosis as it progresses slower in rats than in mice with different phases of immune response.

## Discussion

To our knowledge, this is the first report demonstrating that Cpf1 is applicable to manipulating rat genome by targeting single or multiple loci. Despite advantages over mice and physiological similarity to humans^2^, rats have several limitations compared to mice, such as less robust embryonic stem (ES) cells, entailing reduced options for gene targeting in rats^42^. Engineered nucleases, such as ZFNs, TALENs, and CRISPR/Cas9, have been developed at an exponential pace, enabling us to explore their application for genome editing in rats^6–9^. Recently, Cpf1 was identified as a class 2/type V CRISPR-Cas endonuclease that is distinct from Cas9^10^ and can thus be used as a unique tool or as an alternative when Cas9 cannot be applied. We previously reported genome modification in mice using Cpf1^30^, and here we successfully extended this efficient system to modify the rat genome.

Despite immense efforts for curing atherosclerosis, the research on its early stages in humans has not yet been advanced. This is because human atherosclerosis develops very slowly with individual variations, making it difficult to distinguish between its initiation and progression^29^; moreover, there has been no good animal model to understand the initiation of atherosclerosis. There have been a few studies about *Apoe* knockout rats as atherosclerosis model generated via TALEN^26^ or CRISPR/Cas9^28^, indicating that *Apoe* knockout rat is more resistant to hyperlipidemia-induced endothelial injury than mouse; however, it is also indicated that if fed with HFD for longer period of time and given the atherogenic event, such as ligation of artery, *Apoe* knockout rats would develop atherosclerosis. Considering the progression rate and susceptibility of human atherosclerosis, which takes from months to years or even decades to develop^43^, it could be speculated that *Apoe* knockout rats are more relevant to humans or that they are more appropriate than mice to study the initial stage of atherosclerosis in humans, shifting our focus from progression of atherosclerosis to its initiation, as was partly demonstrated in this study.

The immune system is highly involved in the pathogenesis of atherosclerosis^44^. In the past, most of the atherosclerosis research has focused on intimal accumulation of inflammatory cells^45^ because the initiation of atherosclerosis has traditionally been explained by monocyte adhesion from systemic blood flow to intima (“inside-out”)^33^. However, recently, adventitia has drawn researchers’ attention because vascular inflammation is considered to start within the adventitia and progress toward intima (“outside-in”)^46^. The adventitia is now recognized as a dynamic location that participates in the repair and growth of arterial wall and contains resident cell population, including fibroblasts, lymphocytes, macrophages, and even their progenitor cells^47^, such as AMPCs^41,48^. Furthermore, it has been shown that inflammation in the adventitia is an important early event that correlates with the development of human atherosclerosis^32,49,50^, emphasizing the importance of adventitial contribution to atherosclerosis; however, it still remains unclear how the local immune reaction begins in atherosclerosis. In this study, using *Apoe*-deficient rats, the initial stage of atherosclerosis even before intimal thickening showed adventitial immune infiltrates comprising T lymphocytes and mainly macrophages, and its adventitial source AMPCs were more abundant in rats than in mice, which is first described in this study. Most importantly, this distribution pattern of adventitial immune cells in *Apoe*-deficient rats was in agreement with a previous report on human early stage type I atherosclerosis, wherein it was reported that macrophages comprise the majority of immune cell population in the adventitia, followed by T cells, and that B cells are scarcely found^49^, suggesting the possibility that *Apoe*-deficient rats reflect the immune response of atherosclerosis at initial-to-early stage.

In summary, we successfully generated genetic knockout rats using Cpf1 with targeting *Apoe* and *Ldlr* genes as test cases. It enabled highly efficient single or multiple gene modifications, effective genetic heritability, and minimal off-target effects in rats. The resulting *Apoe* knockout rats will be a better genetically modified animal model for understanding the initiation of human atherosclerosis and further developing preventive or curative medications of cardiovascular disease. Cpf1 is now expanding the range of genome-modifying applications, and we expect that Cpf1-mediated knockout rat models will serve as useful genetic tools for functional genomic research of human diseases.

## Methods

### Generation of crRNAs and *Cpf1* mRNAs

Rat genomic DNA sequences were analyzed and target sequences were selected using the web tool Benchling (https://benchling.com/). crRNAs and *Cpf1* mRNAs were prepared using oligomers listed in Supplementary Table S5 together with pcDNA3.1-hLbCpf1 (for *LbCpf1* mRNA; Addgene #69988) or pcDNA3.1-hAsCpf1 (for *AsCpf1* mRNA; Addgene #69982) obtained from Addgene (http://www.addgene.org/), as previously described^10,51^.

### Microinjection of RNAs into rat embryos and their transfer into foster mothers

All animal experiments were performed in accordance with the Korean Ministry of Food and Drug Safety (MFDS) guidelines. Protocols were reviewed and approved by the Institutional Animal Care and Use Committees (IACUC) of the Asan Institute for Life Sciences (Permit Number: 2015-13-207), and all rats were maintained in the specific pathogen-free (SPF) facility of the Laboratory of Animal Research in the Asan Medical Center (AMClar). To generate mutant rats, Sprague–Dawley (SD) rats were purchased from OrientBio (Gyeonggi, Republic of Korea) for the preparation of embryo donors and foster mothers. Female SD rats (5-week-old) were super-ovulated by intraperitoneal injections of 30-IU pregnant mare serum gonadotropin (PMSG; Sigma) and 100-IU human chorionic gonadotropin (hCG; Daesung microbiological labs Co., Ltd., Gyounggi, Republic of Korea) at 50-h intervals. The super-ovulated female rats were mated to stud males (12-week-old) and checked for the presence of a post-coital vaginal plug the next morning, after which fertilized embryos were collected from the oviducts. Subsequently, *Cpf1* mRNA and crRNA were co-microinjected into the cytoplasm of the pronuclear stage embryos. Normally, 100-ng/μL crRNA and 50-ng/μL *Cpf1* mRNA were co-injected to target the *Apoe* or *Ldlr* genes, and when investigating the dose-dependent effects of crRNA, the concentration of crRNA was decreased to 50 ng/μL. After incubation for 1 hour at 37 °C, the surviving embryos were transferred into the oviducts of synchronized pseudo-pregnant foster mothers.

### Genotyping and sequence analyses

Genomic DNA samples were prepared from tail biopsies to screen for founder rats with potential targeted mutations in *Apoe* or *Ldlr*. Polyacrylamide gel electrophoresis (PAGE)-based genotyping assays were performed as previously described^52^. Briefly, the genomic region that encompassed the target site was PCR-amplified, melted, and re-annealed to form heteroduplex DNA, which was resolved by PAGE. The *Apoe* gene was genotyped by conducting primary PCR reactions using the oligomers 5′-aacagactccacgactgact-3′ and 5′-taggtaggtgcccagatagga-3′, followed by a nested PCR reaction using 5′-ctgttgcttcaaagagacccaag-3′ and 5′-ctggacctggtcagaaagcgt-3′, which would amplify 346-bp and 200-bp DNA fragments, respectively, from the WT allele. The following primers were used in the primary and successive nested PCR reactions for genotyping the *Ldlr* gene: 5′-cgttggtacaggagtggctt-3′ and 5′-ccttaccgcagttctcctcg-3′ (587 bp) and 5′-gagtaggctggtgtgtggtg-3′ and 5′-gggatgcaggaagaggagtt-3′ (242 bp). The PCR products from the *Apoe* and *Ldlr* mutant rats were cloned for sequence analysis using a T-Blunt PCR Cloning Kit (SolGent Co., Ltd., Daejeon, Republic of Korea), and mutations were identified by Sanger sequencing analyses of 15–20 colonies per founder (Macrogen Inc., Seoul, Republic of Korea).

### Western blot analysis

WT and *Apoe*-homozygous knockout rats were generated by intercrossing *Apoe*-heterozygous knockout rats (F_1_) and were subsequently sacrificed at six weeks of age to obtain tissue samples from the brain, liver, kidney, and spleen. Tissue lysates were prepared and analyzed by immunoblot using specific primary antibodies for apolipoprotein E (EPR19392, ab183597, Abcam, Cambridge, UK) and β-actin (C4, sc-47778, Santa Cruz, CA, USA).

### Selection and analysis of off-target sites

Potential off-target sites were selected using Benchling (https://benchling.com/). On-target and potential off-target sites were amplified by nested PCR (Supplementary Table S4). Whether candidate off-target sites were mutated was determined using a PAGE-based genotyping method^52^.

### Analysis of serum cholesterol and lipoproteins

WT and *Apoe*-homozygous knockout rats or mice (8–10 weeks old) were fed with a high-fat diet (HFD; D12336, Research Diets, Inc., NJ, USA) for two weeks, and the serum levels of total cholesterol, high-density lipoprotein, and low-density lipoprotein were measured using a blood biochemical analyzer (Hitachi 7180, Hitachi, Tokyo, Japan).

### Macroscopic and microscopic evaluation of the accumulation of atherogenic components

WT and *Apoe*-homozygous knockout rats or mice (8–10 weeks old) had undergone partial ligation of the left carotid artery (LCA) via sham surgery of right carotid artery (RCA) wherein three of the four caudal branches of the LCA (left external carotid, internal carotid, and occipital artery) were ligated as previously described^34^. Starting immediately after the partial ligation, the rats or mice were fed with HFD for 2 weeks and were subsequently euthanized to collect tissue samples of the LCA, RCA, and entire aorta. The whole aortae were isolated and stained with oil red O (ORO) solution (Sigma) to compare the extent of atherosclerotic lesions among the genotypes, and the percentage of the total ORO-stained lesion area was quantified using NIH software Image J as previously described^53^. For histological studies, fixed tissues were either paraffin embedded, sectioned at 4 μm, and stained with hematoxylin and eosin (H&E) or were embedded in optimum cutting temperature (OCT) compound (Sakura Finetek, CA, USA), cryosectioned at 10 μm, and stained with ORO solution (Sigma).

### Immunofluorescent staining

Paraffin sections were dewaxed, rehydrated, and submitted to heat-induced antigen retrieval in a citrate buffer (10-mM citric acid, 0.05% Tween 20, pH 6.0), and blocked with either 5% goat or donkey serum before incubation with primary antibodies (Supplementary Table S6); this was followed by applying secondary antibodies conjugated to Alexa Fluor (AF) 488, AF 546, or AF 594 (Molecular Probes, Eugene, OR, US). Nuclei were stained with 4′,6-diamidino-2-phenylindole (DAPI) and coverslipped using Dako Fluorescence Mounting Medium (Dako, Santa Clara, CA, US). CD3-, CD19-, and CD68-immunopositive cells were either rarely or not detected in the RCA of both *Apoe*-deficient rats and mice (data not shown). When multiple primary antibodies were combined, false positive cross-reactivity was avoided by choosing primary antibodies raised in different host species and secondary antibodies being applied in two individual steps. For example, rabbit anti-rat Sca1 was used in combination with mouse anti-rat CD45, followed by incubation with AF488 goat anti-rabbit IgG (H + L) for 90 min and subsequent incubation with AF594 goat anti-mouse IgG secondary antibody for 90 min. Microscopy was performed with a Zeiss LSM 780 laser scanning confocal microscope system (Carl Zeiss, Oberkochen, Germany), and the presence of CD45^+^Sca1^+^ AMPCs was confirmed in *Apoe*-deficient rats (Supplementary Fig. S8), as previously described in *Apoe* knockout mice^41^. Since the shape and size of arteries varies in rats and mice, the total immunopositive cell count around the arterial wall was calculated and adjusted by dividing by the mean of the measured long and short diameters between the external elastic lamina (mean diameter, mm) (C/md).

## Supplementary information

Supplementary information
