# Supplementary material for: Knockout rat models mimicking human atherosclerosis created by Cpf1-mediated gene targeting
Source: Sci Rep. 2019 Feb 22;9:2628. doi: 10.1038/s41598-019-38732-2 (PMC6385241; doi:10.1038/s41598-019-38732-2)
Supplement: Supplementary file 1 — Supplementary information [file 41598_2019_38732_MOESM1_ESM.docx]

**Knockout rat models mimicking human atherosclerosis created by Cpf1-mediated gene targeting**

**Jong Geol Lee^1,2,3*^, Chang Hoon Ha^2,4*^, Bohyun Yoon^2^, Seung-A Cheong^1^, Globinna Kim^1,2,4^, Doo Jae Lee^2^, Dong-Cheol Woo^1,2,4^, Young-Hak Kim^5^, Sang-Yoon Nam^3^, Sang-wook Lee^1,6^, Young Hoon Sung^1,2,4^, In-Jeoung Baek^1,2,4^**

^1^ConveRgence mEDIcine research cenTer (CREDIT), ^2^Biomedical Research Center, Asan Institute for Life Sciences, Asan Medical Center, Seoul, Republic of Korea. ^3^College of Veterinary Medicine, Chungbuk National University, Cheongju, Republic of Korea. ^4^Department of Convergence Medicine, ^5^Department of Cardiology, ^6^Department of Radiation Oncology, University of Ulsan College of Medicine, Asan Medical Center, Seoul, Republic of Korea.

*These authors contributed equally to this work.

Corresponding Authors: S.-W.L. ([lsw@amc.seoul](mailto:lsw@amc.seoul).kr), Y.H.S. ([yhsung@amc.seoul.kr](mailto:yhsung@amc.seoul.kr)), and I.-J.B. ([ijbaek@amc.seoul.kr](mailto:ijbaek@amc.seoul.kr)).

**
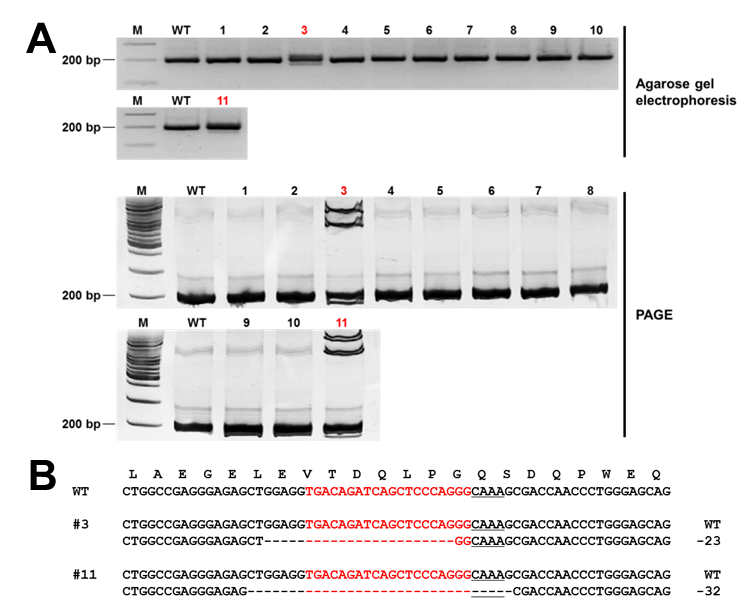
**

**Supplementary Figure S1. Analysis of *in vivo* LbCpf1 activity associated with low-dose crRNA at the rat *Apoe* locus.** Mutant rats were generated by microinjecting *LbCpf1* mRNA (50 ng/μL) and low-dose crRNA (50 ng/μL; shown in Table 1) complementary to a site in the rat *Apoe* locus (shown in Figure 1A). (**A**) The results of agarose gel electrophoresis- and PAGE-based genotyping assays; the numbers indicate individual WT (in black) and mutant (in red) rats. M, molecular size markers. The full-length gels are presented in Supplementary Fig. S11. (**B**) DNA sequences from the tail biopsies of founder rats identified in **A**. The target sequences are shown in red; the underlined sequences represent the PAM and “-” denotes deleted nucleotides. The results are summarized in Table 1.


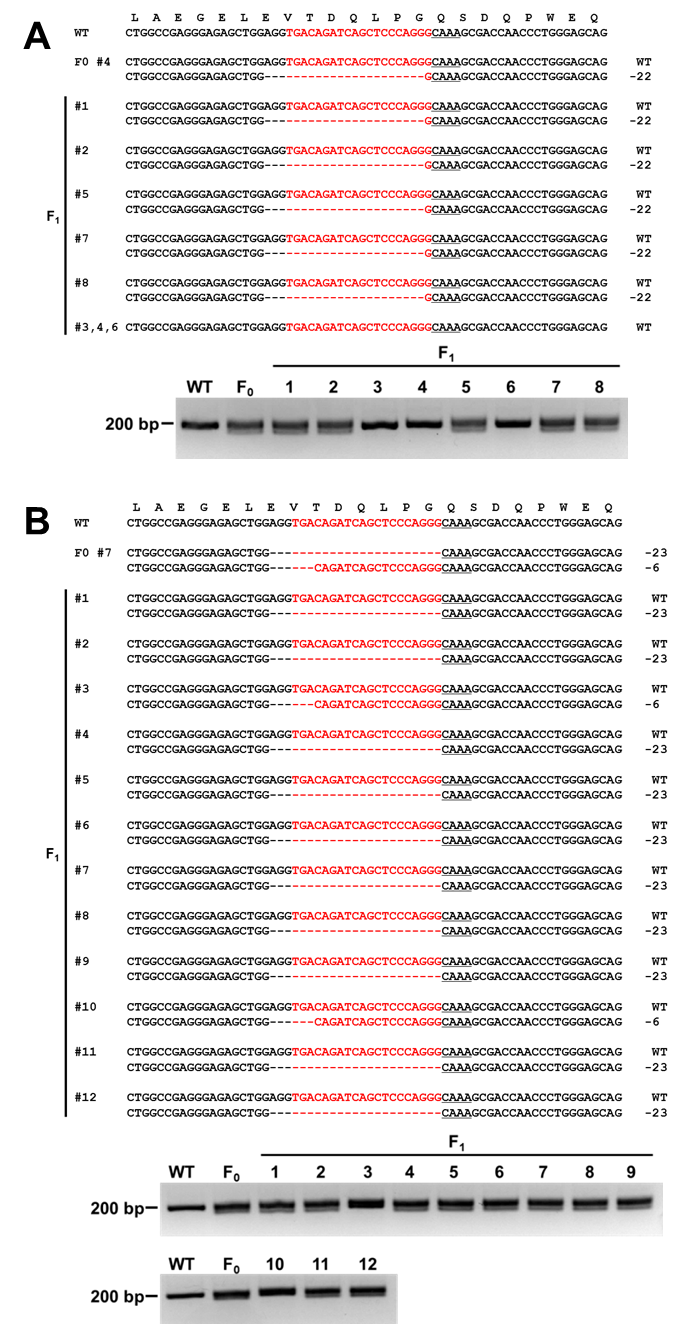


**Supplementary Figure S2. Germ-line transmission of *Apoe* mutant alleles.** *Apoe*-mutant founders #4 (**A**) and #7 (**B**) were crossed with WT rats and the pups’ genotypes were determined by PCR (agarose gel electrophoresis) and subsequent Sanger sequencing. The underlined sequences represent PAM and “-” denotes deleted nucleotides. The full-length gels are presented in Supplementary Fig. S12.


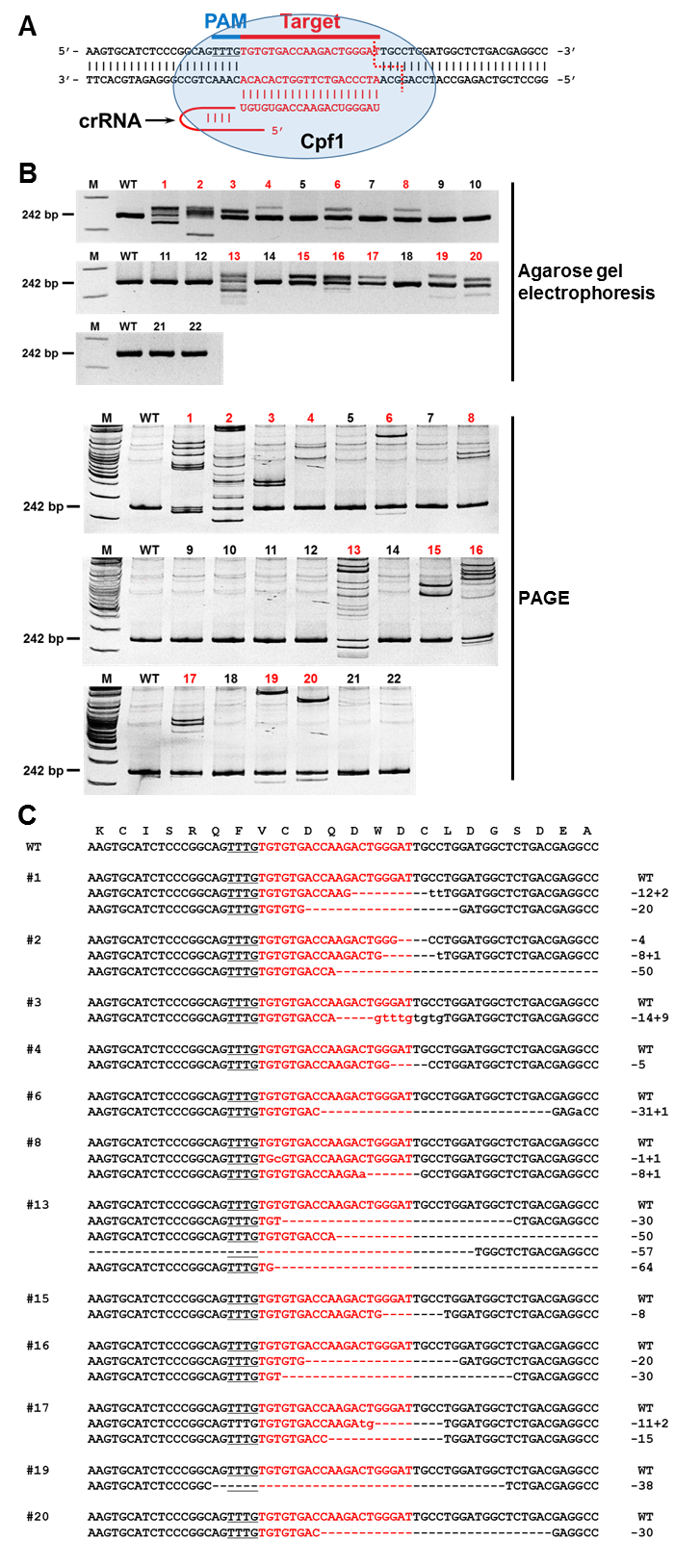


**Supplementary Figure S3. Generation of Cpf1-mediated *Ldlr* mutations in rats.** (**A**) The target DNA sequence in the rat *Ldlr* locus is denoted in red; this region corresponds to the nucleotides 511–576 of the *Ldlr* mRNA (NM_175762.2) encoding amino acids 120–141 of the rat low-density lipoprotein receptor precursor protein (NP_786938.1). The dotted line marks the potential staggered cut that is generated possibly by the Cpf1-crRNA pair and the underlined sequence represents the PAM. (**B**) Agarose gel electrophoresis- and PAGE-based genotyping assays identifying founder rats derived from pronuclear-stage embryos intra-cytoplasmically injected with *AsCpf1* mRNA (50 ng/μL) and its cognate crRNA (100 ng/μL) targeting the rat *Ldlr* locus. The numbers indicate individual WT (in black) and mutant (in red) rats; M, molecular size markers; WT, wild type; *, founder. The full-length gels are presented in Supplementary Fig. S13. (**C**) Mutated *Ldlr* sequences observed in the founders in **B**. “-” denotes deleted nucleotides; underlined sequences represent the PAM; sequences in lower case represent nucleotide substitutions or insertions.

**
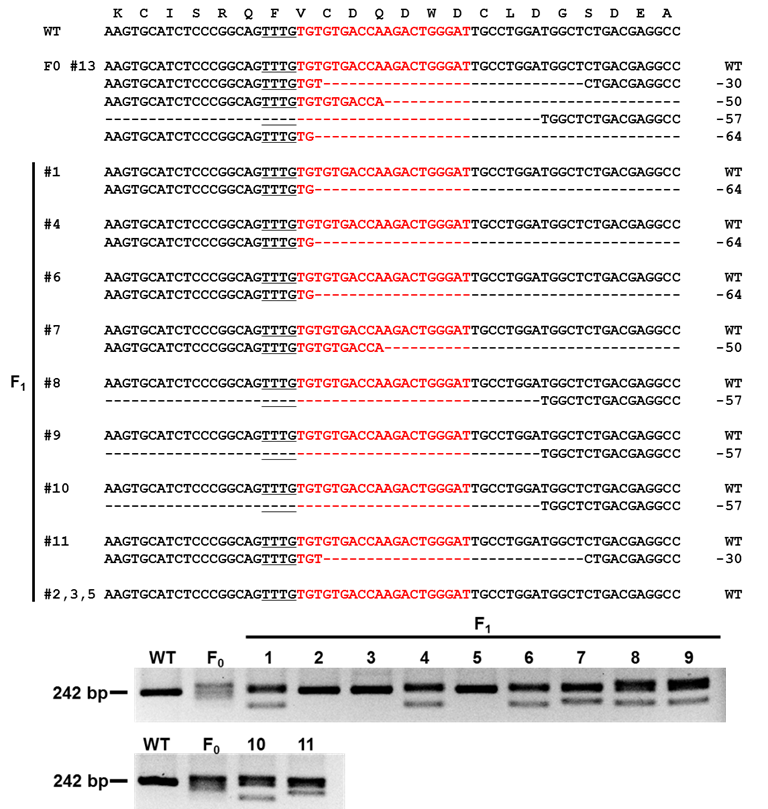
**

**Supplementary Figure S4. Germ-line transmission of the *Ldlr* mutant alleles of founder rats.** *Ldlr*-mutant founder #13 (WT/∆30/∆50/∆57/∆64) generated with AsCpf1 was crossed with WT, and the pups’ genotypes were determined by PCR (gel image) and subsequent Sanger sequencing. The underlined sequences represent the PAM and “-” denotes deleted nucleotides. The full-length gels are presented in Supplementary Fig. S14.


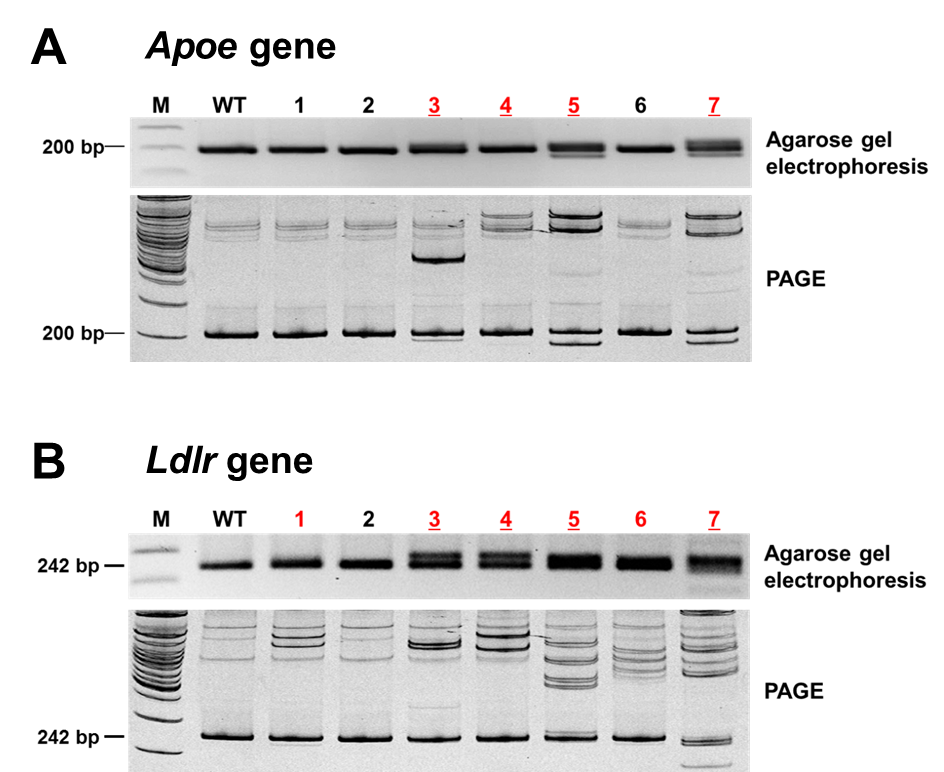


**Supplementary Figure S5. Simultaneous targeting of *Apoe* and *Ldlr* loci using LbCpf1 and AsCpf1, respectively.** *LbCpf1* mRNA (50 ng/μL) paired with crRNA (100 ng/μL) targeting the *Apoe* gene (Fig 1A) and *AsCpf1* mRNA (50 ng/μL) paired with a crRNA (100 ng/μL) targeting the *Ldlr* locus (S3A Fig) were co-injected into zygotes from SD rats. The embryos were isolated from foster mothers at 12–14 days after transfer. (**A, B**) Agarose gel electrophoresis- and PAGE-based genotyping assays screened for mutations in the *Apoe* (**A**) and *Ldlr* (**B**) loci. The non-underlined numbers in red denote embryos with mutations in single genes, the underlined numbers in red represent embryos with mutations in both genes, and the numbers in black indicate WT embryos. M, molecular size markers. The full-length gels are presented in Supplementary Fig. S15. The results are summarized in Supplementary Table S2.


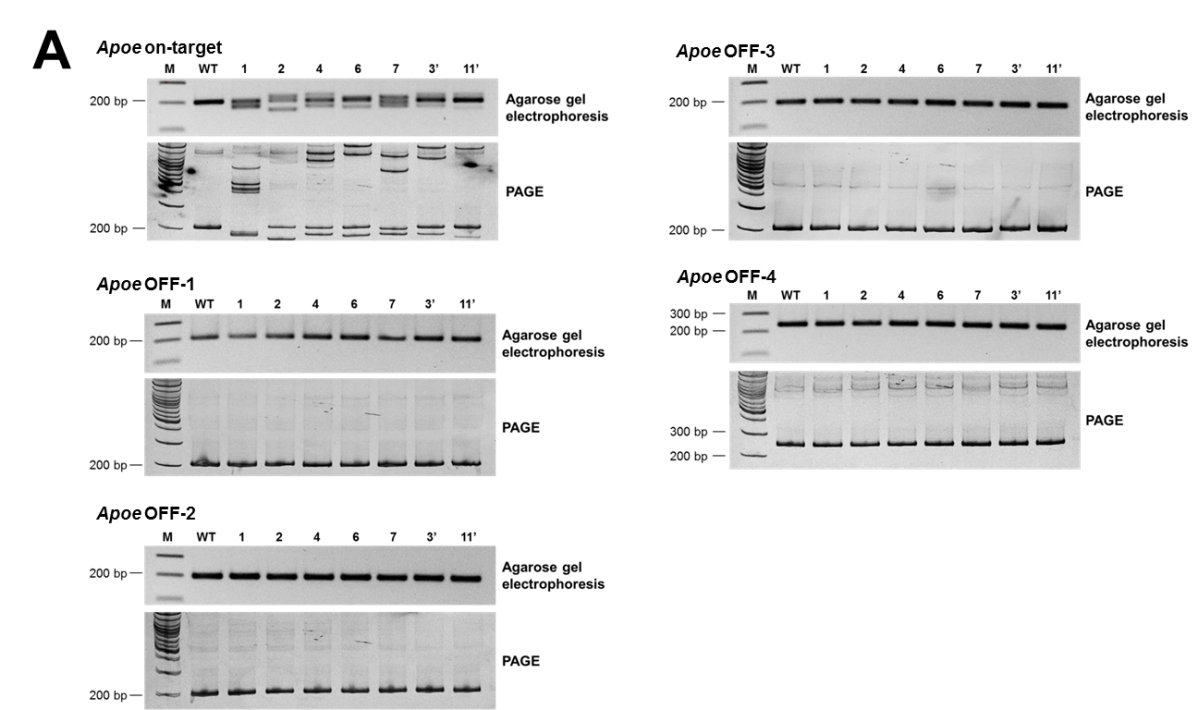


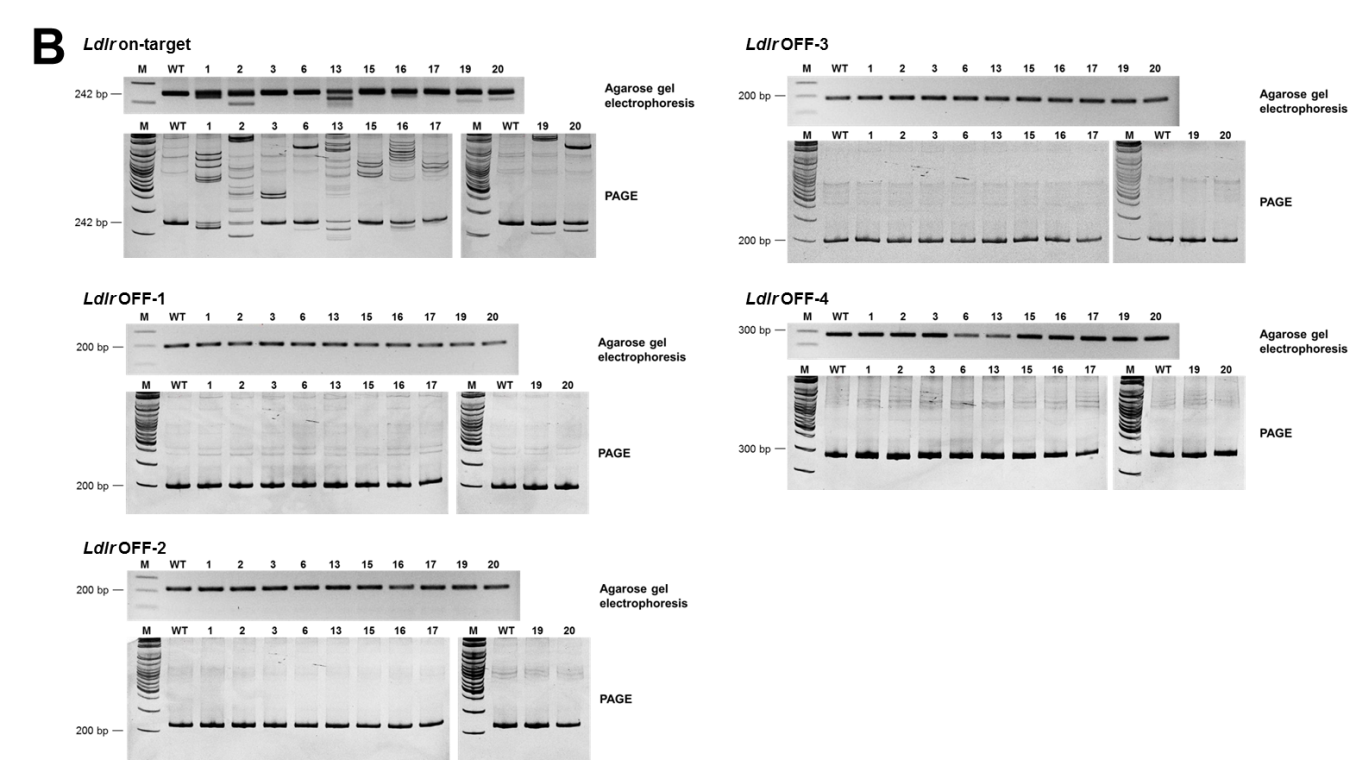


**Supplementary Figure S6. Analysis of potential Cpf1-associated off-target effects in *Apoe* and *Ldlr* mutant founders.** On-target and four potential off-target sites for *Apoe* (**A**) and *Ldlr* (**B**) target regions were amplified from genomic DNA samples from the WT and mutant founder rats by PCR and were then analyzed by agarose gel electrophoresis- and PAGE-based genotyping assays. M, molecular size markers. The full-length gels in Supplementary Fig. S6A and S6B are presented in Supplementary Fig. S16 and S17, respectively.


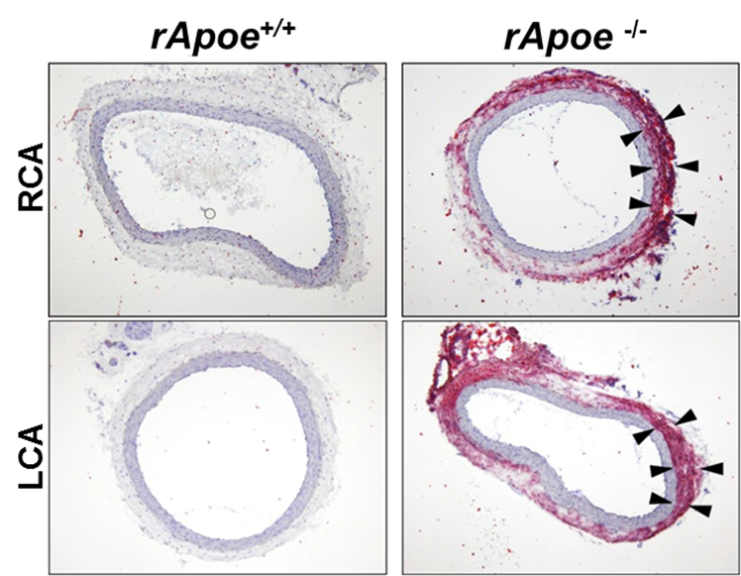


**Supplementary Figure S7. Lipid accumulation in adventitial layers of high-fat-diet fed *Apoe* knockout rats.** After the partial ligation of the left carotid artery (LCA), *Apoe* WT and knockout rats were fed a high-fat diet (HFD) for two weeks, and frozen sections of the LCA and sham right carotid artery (RCA) were stained with oil red O (ORO). Arrowheads indicate adventitial layers exclusively stained with ORO in *Apoe*-deficient carotid arteries. Magnification, ×100.


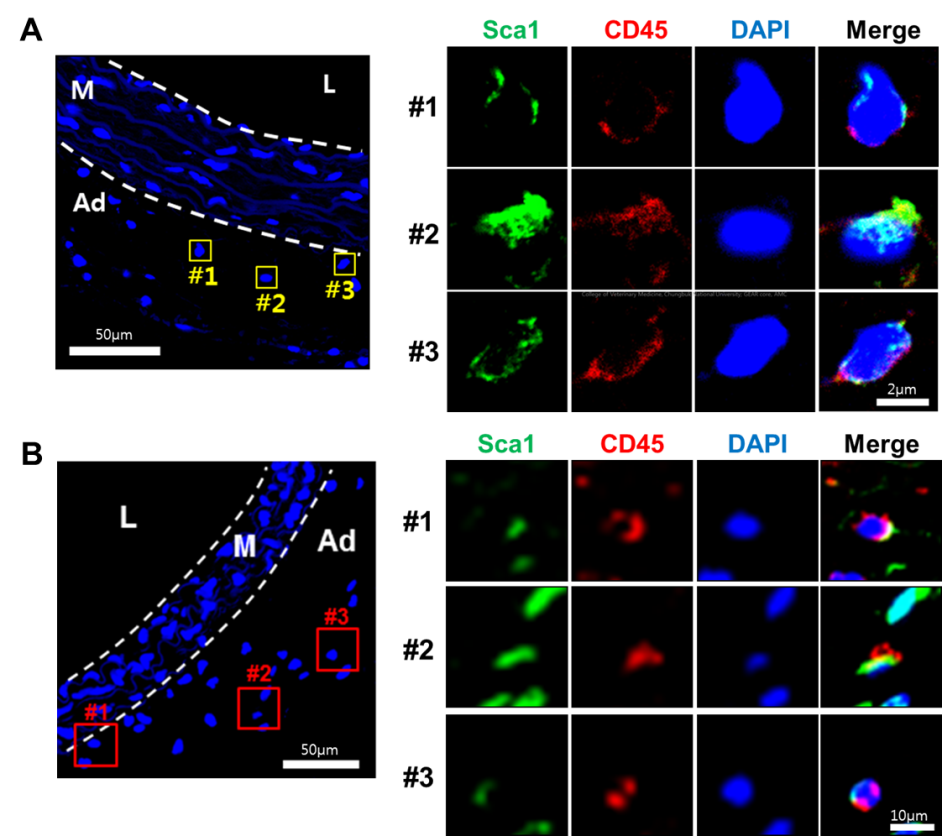


**Supplementary Figure S8. Presence of adventitial macrophage progenitor cells (AMPCs) in the carotid artery of *Apoe* knockout rats and mice.** CD45^+^Sca1^+^ cells were detected in adventitial layers of carotid arteries from *Apoe*-deficient rat (A) and mouse (B) using a confocal microscope. L, lumen of carotid artery; M, media; Ad, adventitia.

**Supplementary Table S1.** AsCpf1-mediated *Ldlr* gene targeting in Sprague–Dawley rat embryos.

| crRNA +  *Cpf1* mRNA (ng/μL) | Injected  zygotes | Survived & transferred (%)^a^ | Newborns (%)^b^ | Mutants (%^b^, %^c^) |
| --- | --- | --- | --- | --- |
| 100 + 50 | 63 | 63 (100) | 22 (34.9) | 12 (19.0, 54.5) |

Percentages were calculated using the number in each column as the numerator and the numbers of ^a^injected zygotes, ^b^surviving and transferred embryos, or ^c^newborns as the denominator.

**Supplementary Table S2.** Simultaneous gene targeting at the *Apoe* locus using LbCpf1 and the *Ldlr* locus using AsCpf1 in SD rats.

| LbCpf1+*Apoe-*crRNAs, AsCpf1+ *Ldlr*-crRNA (ng/μL) | Injected zygotes | Survived zygotes (%)* | | E12.5–14.5  Embryos  (%)† | Mutants (%†, %††) | | |
| --- | --- | --- | --- | --- | --- | --- | --- |
|  |  |  |  |  | *Apoe* | *Ldlr* | *Apoe; Ldlr* |
| 100 + 50,  100 + 50 | 113 | 113  (100) | 7  (6.2) | | 4  (3.5, 57.1) | 6  (5.3, 85.7) | 4  (3.5, 57.1) |

Percentages were calculated using the number in each column as the numerator and the number of *injected zygotes, †surviving and transferred embryos, or ††isolated E12.5–E14.5 embryos as the denominator.

**Supplementary Table S3.** Potential off-target sites that contain 3- to 4-bp mismatches relative to target sequences;
mismatches are written in lower case.

| **Target** | **OFF-target No.** | **DNA** | **Chr.** | **Position** | **Direction** | **Mismatches (bp)** |
| --- | --- | --- | --- | --- | --- | --- |
| ***Apoe*** | **On-target** | **CCCTGGGAGCTGATCTGTCA** | **chr1** | **80614268** | **+** | **0** |
|  | OFF-1 | CCCTGGGgGaTGATCTGTgA | chr3 | 54566100 | - | 3 |
|  | OFF-2 | CtCTGGGAGCaGATCTtTCc | chr9 | 20112912 | + | 4 |
|  | OFF-3 | CtCTGGGAGtTGATCTcTaA | chr2 | 181297559 | + | 4 |
|  | OFF-4 | tCCTGGGAGCTGATCacTgA | chr7 | 24165138 | - | 4 |
| ***Ldlr*** | **On-target** | **TGTGTGACCAAGACTGGGAT** | **chr8** | **22759662** | **+** | **0** |
|  | OFF-1 | TGTcTGAaCAAGACTGGGtT | chr8 | 127376434 | + | 3 |
|  | OFF-2 | TGTGTGACCAAcACTGGGca | chr19 | 51130429 | - | 3 |
|  | OFF-3 | TGTGTGACCAgtACTGGcAg | chr17 | 28704949 | - | 4 |
|  | OFF-4 | TGTGTGACCAtGACcGcGAc | chr7 | 70864278 | - | 4 |

**Supplementary Table S4.** List of primers used for PCR reactions to analyze off-target effects.

| **Target** | **OFF-target No.** | **1st F** | **1st R** | **bp** | **2nd F** | **2nd R** | **bp** |
| --- | --- | --- | --- | --- | --- | --- | --- |
| *Apoe* | On-target | AACAGACTCCACGACTGACT | TAGGTAGGTGCCCAGATAGGA | 346 | CTGTTGCTTCAAAGAGACCCAAG | CTGGACCTGGTCAGAAAGCGT | 200 |
|  | OFF-1 | AGTACTTTTGGAAGAATTCACAGC | AGATGCATAGTTAGCCGCCG | 487 | TCACATGACCTCAGGGCAGA | ACTCTGTCGCTCGCTCTTC | 200 |
|  | OFF-2 | CCTTTTGCCTCTGTTCATAGTTCT | GTCTTGTGTATGTTTTGGCGCT | 600 | TTGTTGACAGCGTTGAGATTATCG | GGAGAAAAGGGCCTGTGGTAATA | 198 |
|  | OFF-3 | TTCTGGTCACTCACTCACTCACTAA | CAATGGTTCCCAAGTTGTTTCTGA | 457 | AGAAGTGGAGACCTTTGCCTT | TGTCAATCATTAGCTCTTTCTGACT | 194 |
|  | OFF-4 | TCTGTCCTGCCAGTAGGGATT | CTCCCTGATCTAGTGCCATTAAGAA | 497 | CTCCTATGGCTTAGCGGGTCA | AGTCACACCCCAAGATTACGTT | 233 |
| *Ldlr* | On-target | CGTTGGTACAGGAGTGGCTT | CCTTACCGCAGTTCTCCTCG | 587 | GAGTAGGCTGGTGTGTGGTG | GGGATGCAGGAAGAGGAGTT | 242 |
|  | OFF-1 | TCTGTGGCTGTAGGGATGGA | TGGAATTCCCAACCGCCTTT | 496 | CCTACACTTTGCTTTGTTTCCCTC | CACTCTGCACAGTAGCCCGA | 193 |
|  | OFF-2 | TCCCCTTGTAGGAGAAGGCA | CCAGGTCTGGCTCCTTGATG | 950 | GGAACCTCTACTAGCTCGCAAT | CCTGTTAAACAACCGGACCC | 218 |
|  | OFF-3 | ACCTCCCTGTACAATGAAAAACA | TGGCAGTTCTTTCTATAACATTGC | 694 | TCCTAACTCCCTCCTAAAGAGT | AAAGCTCTCTTCTGGACACCT | 184 |
|  | OFF-4 | GGGTTCTTGGGAGCCTCATC | ACTCCTCCCATTCTGTCCGA | 465 | GGGTATTCTGTGGTGGGGAG | TAGCTAGTAGGTCAGGGCTGG | 277 |

**Supplementary Table S5.** List of primer sequences used in crRNA preparation.

| **Primers** | **Sequence (5′ to 3′)** |
| --- | --- |
| T7 top strand primer | GAAATTAATACGACTCACTATAGGG |
| Anti-T7-LbApoe | TGACAGATCAGCTCCCAGGGATCTACACTTAGTAGAAATTACCCTATAGTGAGTCGTATTAATTTC |
| Anti-T7-AsLdlr | ATCCCAGTCTTGGTCACACAATCTACAAGAGTAGAAATTACCCTATAGTGAGTCGTATTAATTTC |

**Supplementary Table S6.** List of antibodies and reagents used in immunofluorescence staining.

| **Antibody** | **Manufacturer** |
| --- | --- |
| ***Primary antibodies*** |  |
| Rabbit anti-rat Sca1 | Millipore, Temecula, CA, US |
| Mouse anti-rat CD45 | BD Pharmingen |
| Goat anti-mouse Sca1 | R&D Systems, Minneapolis, MN, US |
| Rat anti-mouse CD45 | BD Pharmingen, San Jose, CA, US |
| Rabbit anti-CD68 | Abcam, Cambridge, UK |
| Rabbit anti-CD3 | Dako, Santa Clara, CA, US |
| Rat anti-CD19 | ThermoFisher Scientific, Waltham, MA, US |
| ***Secondary antibodies*** |  |
| AF488 goat anti-rabbit | Molecular Probes, Eugene, OR, US |
| AF594 goat anti-mouse | Molecular Probes |
| AF488 donkey anti-goat | Molecular Probes |
| AF594 donkey anti-rat | Molecular Probes |
| AF546 donkey anti-rabbit | Molecular Probes |
| ***Others*** |  |
| Donkey serum | Jackson Laboratory, Bar Harbor, ME, US |
| Goat serum | Vector Laboratories, Burlingame, CA, US |
| DAPI | Molecular Probes |


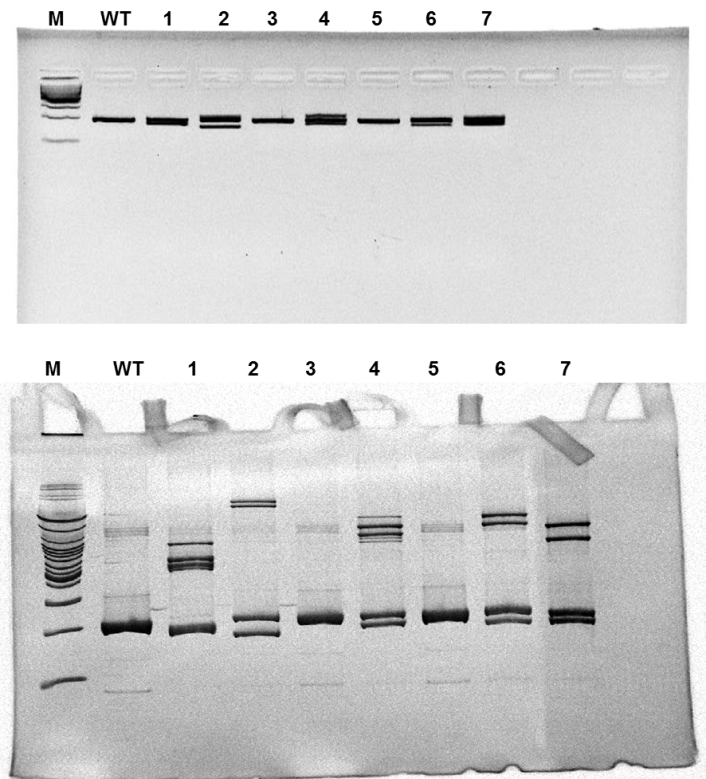


**Supplementary Figure S9. Full-length gels of Figure 1B.**


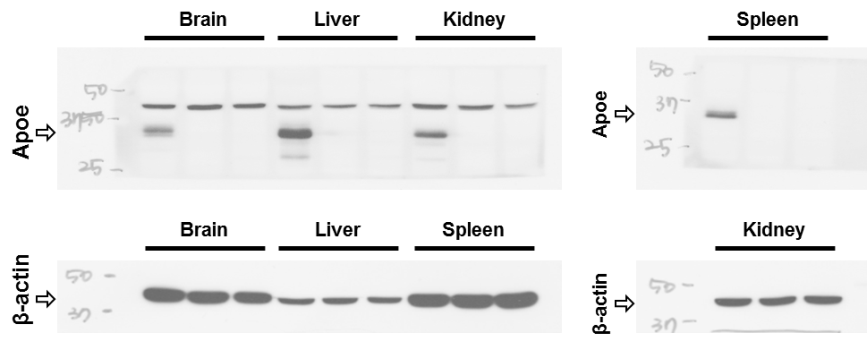


**Supplementary Figure S10. Full-length blots of Figure 1D.**


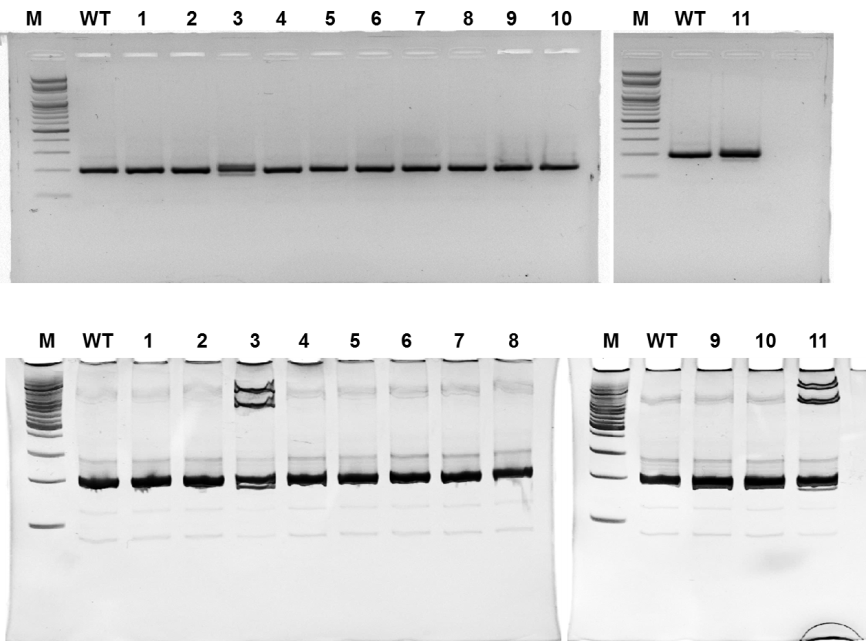


**Supplementary Figure S11. Full-length gels of Supplementary Figure S1A.**


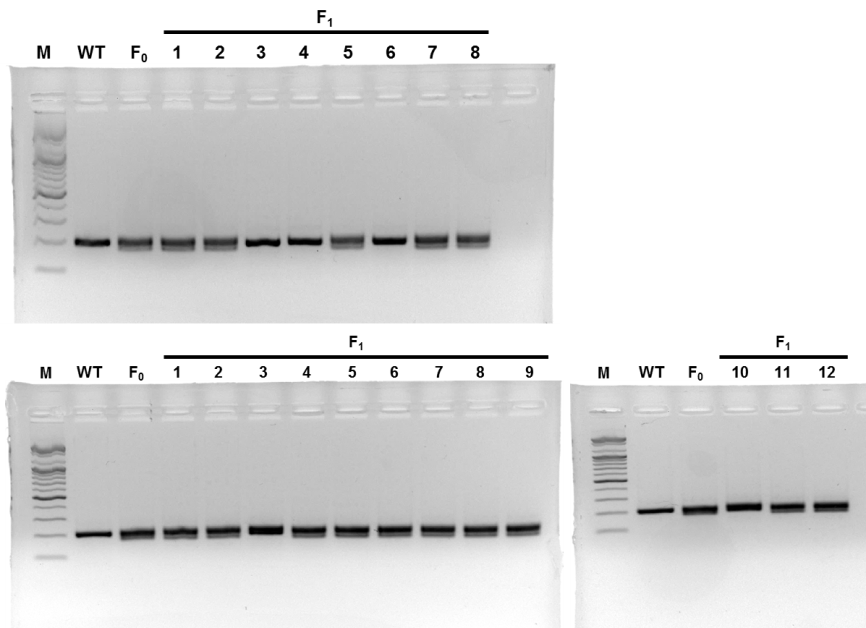


**Supplementary Figure S12. Full-length gels of Supplementary Figure S2A (upper panel) and S2B (lower panel).**


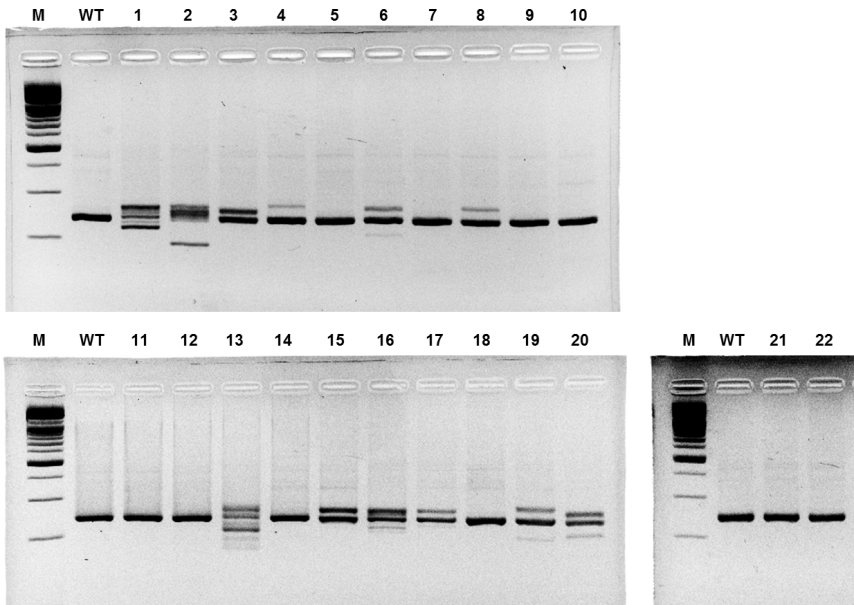


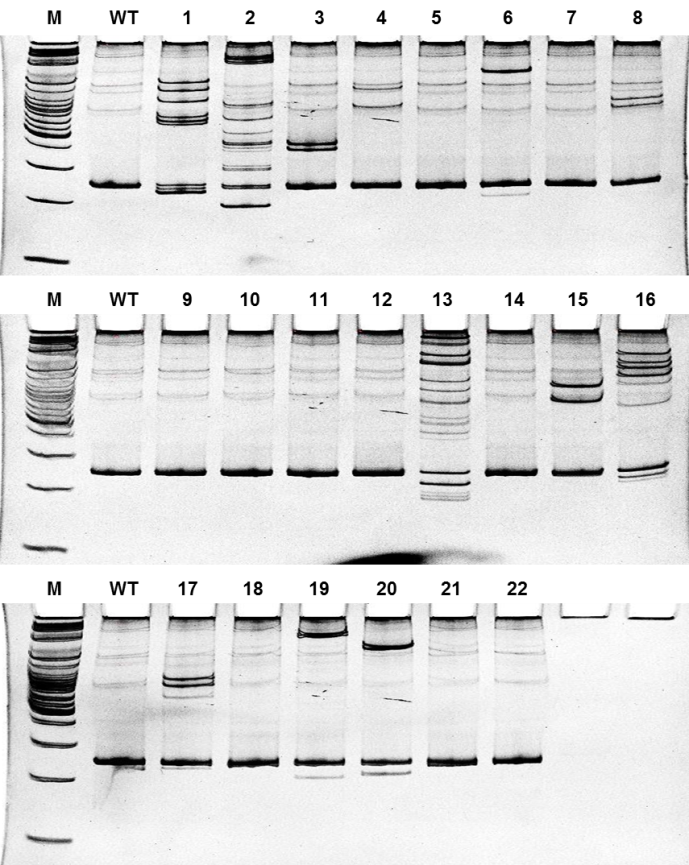


**Supplementary Figure S13. Full-length gels of Supplementary Figure S3B.**


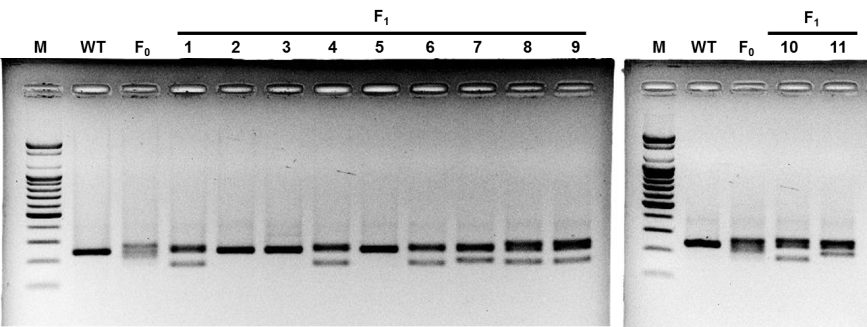


**Supplementary Figure S14. Full-length gels of Supplementary Figure S4.**


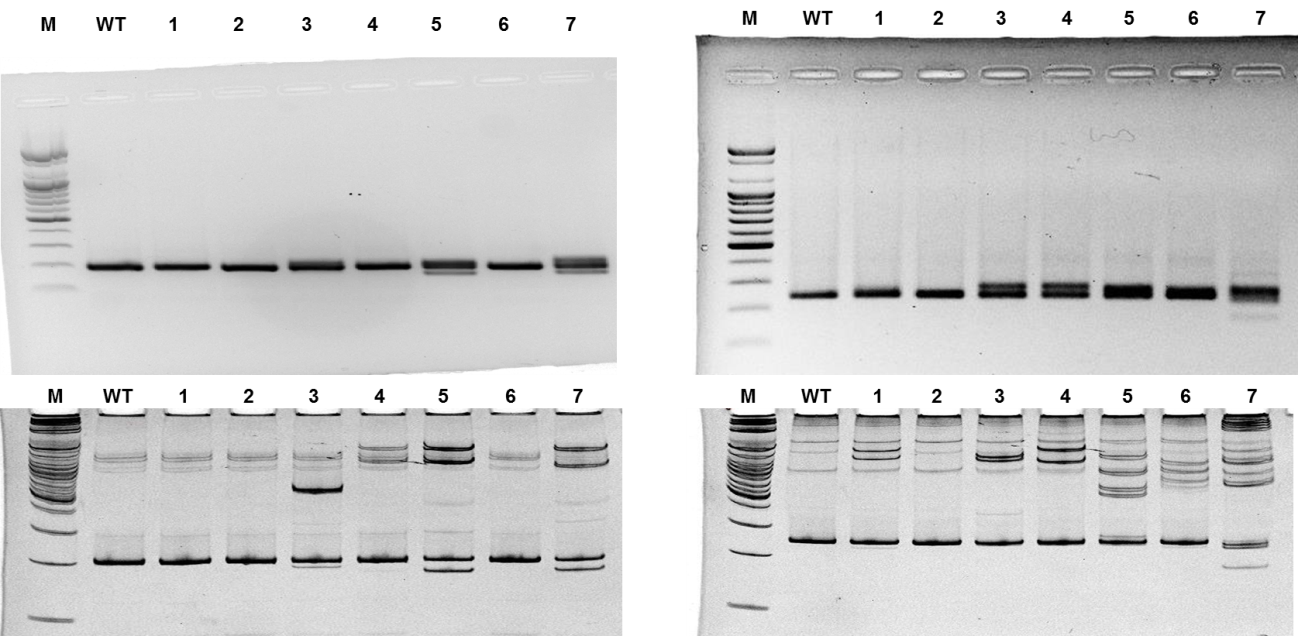


**Supplementary Figure S15. Full-length gels of Supplementary Figure S5A (left panel) and S5B (right panel).**


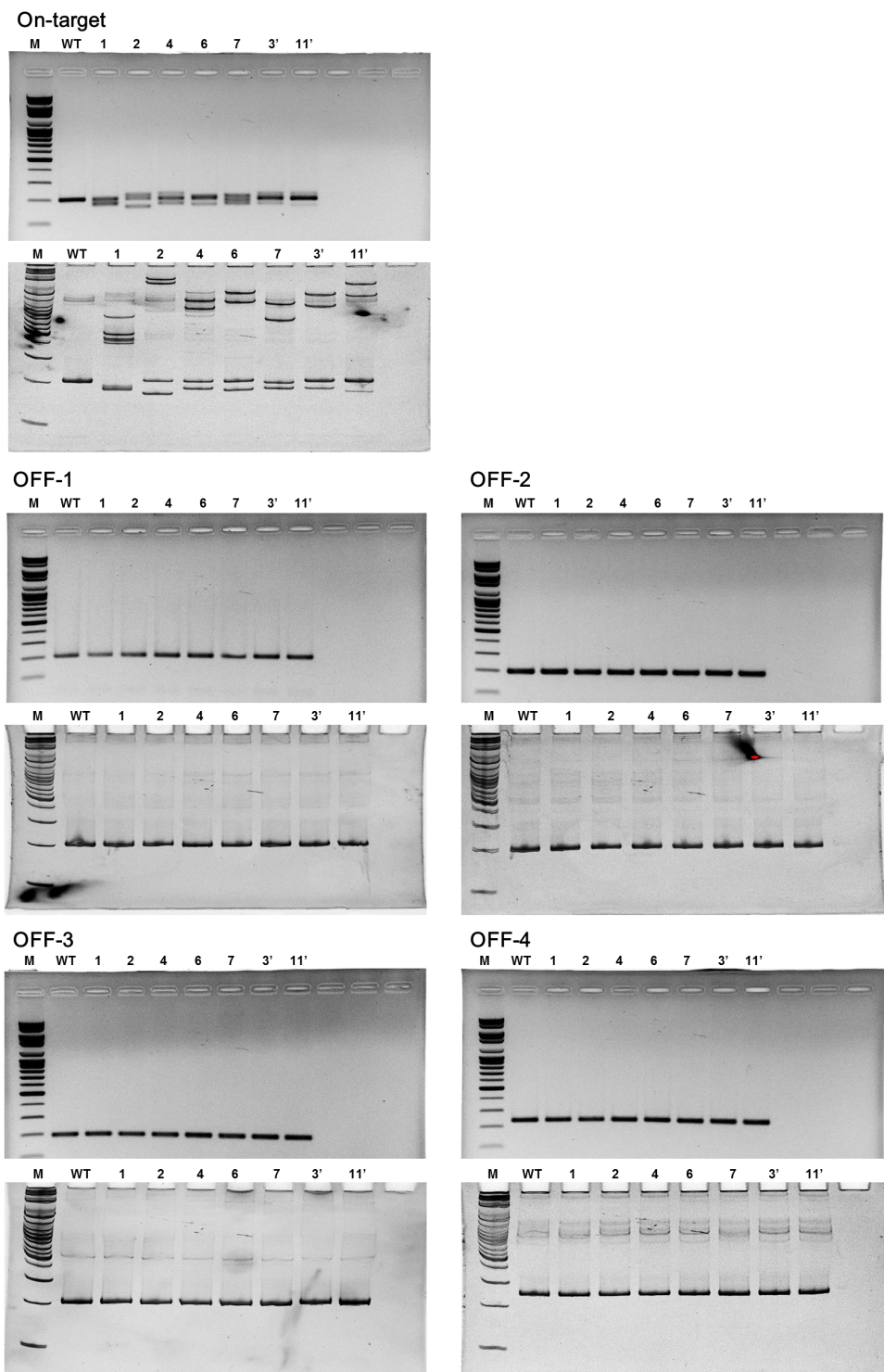


**Supplementary Figure S16. Full-length gels of Supplementary Figure S6A.**

**
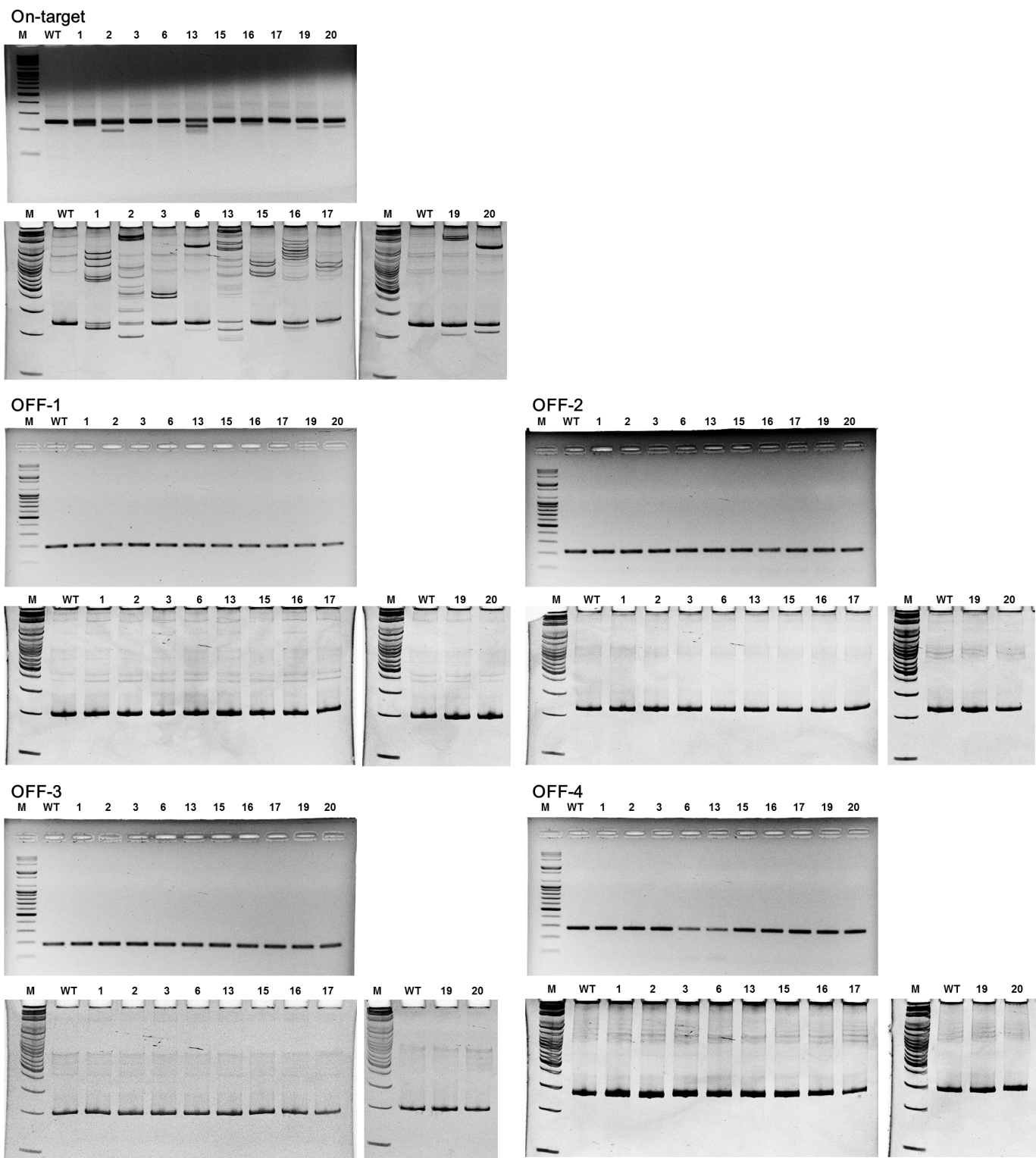
**

**Supplementary Figure S17. Full-length gels of Supplementary Figure S6B.**
